# Time estimation during motor activity

**DOI:** 10.3389/fnhum.2023.1134027

**Published:** 2023-04-21

**Authors:** Ottavia D’Agostino, Serena Castellotti, Maria Michela Del Viva

**Affiliations:** Department of Neurofarba, University of Florence, Florence, Italy

**Keywords:** duration estimation, walking, motor load, motor-cognitive interference, time estimation

## Abstract

Several studies on time estimation showed that the estimation of temporal intervals is related to the amount of attention devoted to time. This is explained by the scalar timing theory, which assumes that attention alters the number of pulses transferred by our internal clock to an accumulator that keeps track of the elapsed time. In a previous study, it was found that time underestimation during cognitive-demanding tasks was more pronounced while walking than while sitting, whereas no clear motor-induced effects emerged without a concurrent cognitive task. What remains unclear then is the motor interference itself on time estimation. Here we aim to clarify how the estimation of time can be influenced by demanding motor mechanisms and how different motor activities interact with concurrent cognitive tasks during time estimation. To this purpose, we manipulated simultaneously the difficulty of the cognitive task (solving arithmetic operations) and the motor task. We used an automated body movement that should require no motor or mental effort, a more difficult movement that requires some motor control, and a highly demanding movement requiring motor coordination and attention. We compared the effects of these three types of walking on time estimation accuracy and uncertainty, arithmetic performance, and reaction times. Our findings confirm that time estimation is affected by the difficulty of the cognitive task whereas we did not find any evidence that time estimation changes with the complexity of our motor task, nor an interaction between walking and the concurrent cognitive tasks. We can conclude that walking, although highly demanding, does not have the same effects as other mental tasks on time estimation.

## Introduction

Everyday situations require the ability to perform two or more tasks at the same time, such as walking and using mobile phones, driving and talking, cooking while listening to the news. A large body of evidence shows interactions between motor and cognitive domains. It has been demonstrated that simultaneously performing motor and cognitive tasks leads to the decline of one or both performances [for a review, see (Al-Yahya et al., 2011)]. The most common paradigm for studying motor-cognitive interference is the concurrent performance of hand movements and verbal assignment (Simon and Sussman, 1987; Hiscock et al., 1989; Serrien, 2009; Matheson et al., 2014). For instance, hand movements affect verbal counting performance (Serrien, 2009), visual processing capacity (Künstler et al., 2018), object-naming (Matheson et al., 2014; Koester and Schack, 2016), speech tasks (Dromey and Benson, 2003), and working memory (Spiegel et al., 2013; Logan and Fischman, 2015).

Some studies also used other motor tasks such as maintaining balance or walking [for a review see (Bayot et al., 2018)]. These gross motor functions have traditionally been considered automatic activities that do not require the involvement of cognitive processing in young healthy adults (Paul et al., 2005; Bridenbaugh and Kressig, 2011; Clark et al., 2015). However, this assumption has been challenged by some evidence highlighting that they are complex mechanisms requiring cognitive and attentional processes [(Lajoie et al., 1993; Ebersbach et al., 1995; Woollacott and Shumway-Cook, 2002; Plummer et al., 2015; Pizzamiglio et al., 2017; Herold et al., 2018; Nieborowska et al., 2019), for a review, see (Leone et al., 2017)]. For example, Ebersbach et al. (1995) investigated the effect of different tasks (cognitive, fine motor, and combined tasks) on walking, finding that only combined mental and fine motor demands interfere with the regulation of balance during walking, suggesting that the effect of a concurrent task on gross body movements depended on the difficulty of the secondary task. On the other end, walking and balance control reduce cognitive abilities, such as solving math operations (Chong et al., 2010; Castellotti et al., 2022).

However, most of these studies only used simple motor tasks, using people’s normal speeds and rhythm. Only a few studies investigate the effect of motor task difficulty on cognitive tasks, finding controversial results. For example, Kelly et al. (2010) did not find any influence of walking difficulty on the performance in auditory stroop task. On the contrary, Lindenberger et al. (2000) required participants to perform memory encoding while walking through two tracks with different path complexity, finding that the higher the walking difficulty the stronger the interference with the cognitive performance.

In everyday life, one common cognitive activity that humans perform while walking is perceiving elapsed time. Time perception, defined as the subjective experience of the passage of time, plays a crucial role in human life, and it is affected by many contextual variables (Block, 2003; Block and Zakay, 2008; Wittmann, 2009; Block et al., 2010; Grondin, 2010; Sucala et al., 2010; Merchant et al., 2013; Matthews and Meck, 2014). Humans’ ability to estimate temporal duration has been broadly investigated, mainly using brief time intervals, in the order of 10–100 ms or order of seconds, while a few explored temporal estimations of intervals in the range of minutes [for reviews, see (Buhusi and Meck, 2005; Ivry and Schlerf, 2008; Baldauf et al., 2009; Grondin, 2010; Matthews and Meck, 2016)].

Overall, the literature agrees with the hypothesis that time estimation involves the presence of an internal clock (Gibbon et al., 1984; Wearden, 1999; Buhusi and Meck, 2005). In this view, there is a pacemaker that produces pulses, which are accumulated in a counter, and the number of pulses counted determines the perceived length of an interval (Rammsayer and Ulrich, 2005). According to the scalar expectancy theory (Gibbon, 1977; Wearden, 2003), in addition to the initial clock process, there are two further levels: the memory and the decision-making stage (Meck, 2003). The timing errors are due either to a change in the pacemaker’s rate or to attentional resource allocation to the timing task [*attention allocation model*, (Zakay et al., 1983; Brown and West, 1990; Macar et al., 1994)].

A wide range of research involving dual task paradigms demonstrated a shortening of perceived time with the increasing of the concurrent task difficulty (Brown, 1985; Zakay and Tsal, 1989; Rammsayer and Ulrich, 2005; Castellotti et al., 2022); indeed, as theorized by the *attention allocation model*, paying attention only to time induces temporal overestimation, whereas diverting attention away from time causes time underestimation, with a positive relationship with the difficulty level of the concurrent non-temporal tasks [(Burnside, 1971; Thomas and Weaver, 1975; Macar et al., 1994), for reviews, see (Block et al., 2010; Gu et al., 2015)].

Some behavioral data suggest a more complex framework, as theorized by the working memory model (Baddeley, 2002) and the multiple resource model (Wickens, 2002). For instance, a recent study by Polti et al. (2018) tried to distinguish the role of WM and attention in time estimation testing real human activities duration. They used durations up to 90 s while participants had to attend only to time (single task) or to perform an N-back WM-task, finding a significant underestimation, proportional to WM load, in the dual task condition. Also, Brown (1997) tested temporal reproduction of intervals on the order of seconds while performing three different non-temporal tasks and found that the temporal performance was disrupted by all three cognitive tasks, whereas only mental arithmetic was disrupted by timing (Brown, 1997). This could be explained by the fact that temporal estimation and mental arithmetic compete for the same resources, causing mutual interference (Brown, 1997; Block, 2003).

Only recently, some studies have investigated whether temporal perception can be affected by motor processes, mainly using fine movements. Indeed, many of them tested the effects of hand movements on judging short durations of auditory (Yon et al., 2017) and visual stimuli (Yokosaka et al., 2015; Tomassini and Morrone, 2016), finding that time distortions are linked to the motor system. So far, to our knowledge, only a few studies investigated how time estimation is affected by gross body movement like walking (Kroger-Costa et al., 2013; Sayali et al., 2018; Spapé et al., 2022). Overall, they found overestimation of temporal durations during walking and speculated that movement speeds up the internal clock (Kroger-Costa et al., 2013; Sayali et al., 2018; Spapé et al., 2022).

In our previous study, for the first time, we combined time estimation of long durations (up to 2 min), four different cognitive tasks of increasing difficulty, and two different motor conditions (Castellotti et al., 2022). Our results showed that, when participants were sitting on a chair (absence of motion), they tended to underestimate durations during cognitive-demanding tasks and overestimate durations while attending only to time [in line with the *attentional allocation model* (Grondin, 2010; Allman et al., 2014; Matthews and Meck, 2016)]. Also, estimation uncertainty increased linearly with time estimates in all tasks, in agreement with the scalar timing theory (Buhusi and Meck, 2005; Grondin, 2010; Matthews and Meck, 2014). When participants were walking, estimation bias during mental tasks was more pronounced, as well as estimation uncertainty, whereas no clear motor-induced effects emerged without a concurrent cognitive task. Overall, it seemed that the motor load adds somehow to the cognitive load in distorting temporal judgment, but whether the motor tasks themselves interfere with time estimation remains unclear. We could hypothesize that a demanding walking activity might require a sufficient amount of attention to affect time estimation per se in the absence of another concurrent cognitive task. According to the *scalar timing theory*, this would mean that walking tasks might directly open the switch, causing a loss of pulses and thus decreasing the perceived duration, as well as other demanding cognitive tasks do. In the previous study, we did not observe this effect, maybe due to the relatively automatic walking task used in the experiment.

The exact nature of the interaction between walking activity and other cognitive tasks (e.g., linear or non-linear) and the relative amount of attention dedicated to the two processes still remain unclear. The observed smaller effect exerted by the motor vs. cognitive task suggests a larger amount of attention dedicated to the latter. However, by using just one type of relatively automatic motor activity, we could not deduce the nature and the relative strength of the interaction (Castellotti et al., 2022).

For these reasons, here we manipulate simultaneously the difficulty of the cognitive task, requiring solving mathematical operations of increasing difficulty, and that of the concurrent motor task, testing three different types of walking: forward regular-speed walking, forward irregular-speed walking, and backward irregular-speed walking. We then compare the effects of these three types of walking on time estimation, by measuring accuracy and uncertainty. If the walking activity directly affects time estimation, in the absence of another cognitive task, we expect larger time underestimations and larger uncertainties for backward irregular-speed walking than for forward regular-speed walking. By comparing the effects of different combinations of difficulty levels of the two tasks, it would be also possible to shed some light on the nature and strength of their interactions.

The findings of this work will highlight whether complex body movements can interfere with time estimation and clarify how different motor and cognitive processes interact during time estimation.

## Materials and methods

### Participants

Fifteen young adults (μ = 24.8 years, SE = 0.8) participated in the experiment. All the participants were university students, naïve to the purpose of the study and they had given written informed consent prior to participation. They were also required to possess a valid medical certificate for the physical activity involved. Before data collection, they were asked to fill out a questionnaire regarding personal data, expertise in some specific fields (e.g., sport or music), sleeping habits, presence of any optical damage or pathological disorders (e.g., dyscalculia), medication intake (e.g., psychotropic drugs or sleeping pills). They were also asked to subjectively rate their math-anxiety level and their ability in solving mathematical operations, on a 7-point Likert scale. All participants had normal or corrected-to-normal visual acuity, did not take any type of medication, did not present any brain damage, and were free of cognitive disorders. All reported having a regular sleep-wake cycle (average night sleep duration of 7.5 ± 0.3 h). None of them was a professional athlete or musician and they reported, on average, having good ability in solving mathematical sums (μ = 4.7, SE = 0.4) and low math anxiety (μ = 2.1, SE = 0.3) Participants were asked to wear light sporting clothes and comfortable shoes during the experimental session and to not assume stimulating substances the night before the experiment. Before each experimental session, they were asked to rate their stress level and their mental and physical tiredness, on a 7-point Likert scale.

The study was conducted according to the guidelines of the Declaration of Helsinki and approved by the local ethics committee (“*Commissione per l’Etica della Ricerca”, University of Florence, 7 July 2020, n. 111*”).

### Setup

Participants walked on a JK Fitness treadmill (Supercompact 48 model, 48 × 130 cm walking belt), positioned 80 cm away from the display, that subtended 43° × 24° of visual angle. Stimuli were programmed and displayed on an iMac Retina 5K 27-inch [mid 2015, 3.3 GHz Intel Core i5 processor, MacOs Mojave software 10.12.6 (Cupertino, CA, USA), frame rate 60 Hz, 5,120 × 2,880 pixels resolution]. Participants’ responses were entered on a computer keyboard by the experimenter. The experimenter measured the participants’ head temperature through a non-contact infrared thermometer (Berrcom, JXB-178 model). Software for presentation of stimuli and data collection was developed using the Psychophysics Toolbox extensions for Matlab (R2018b version; Natick, MA, USA: The MathWorks Inc.).

### Procedure and conditions

**FIGURE 1:**
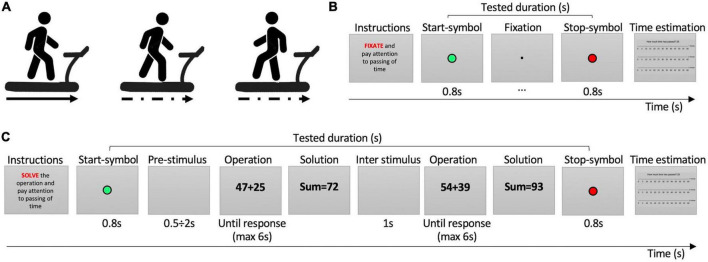
Procedure and tasks. **(A)** Motor conditions. From left to right: forward regular-speed walking, forward irregular-speed walking, backward irregular-speed walking. **(B)** Easy task. **(C)** Hard task. In the instructions, the red word indicates the cognitive task to be performed. The green circle indicates the beginning of time estimation; the red circle indicated the stop of time estimation.

The entire experiment required three sessions of 2 h per participant on three different days, one for each motor condition: (1) forward regular-speed walking, (2) forward irregular-speed walking, and (3) backward irregular-speed walking (Figure 1A). The first is an automated movement performed without any particular effort (Paul et al., 2005; Bridenbaugh and Kressig, 2011; Clark et al., 2015): each participant freely chose the most comfortable walking speed by adjusting it on the treadmill console before starting the session. The average speed chosen is 2.5 ± 0.2(SE) km/h. The second requires some motor control and body balance since the speed changes unpredictably for the walker; indeed, the experimenter randomly changed it every 1–3 s, in the range from 2 to 8 km/h (maximum speed to avoid running). The third is a very unusual movement for humans and can be considered highly demanding because it requires attention to be implemented and continuous motor control to adapt to the random change of speed; indeed, during the session, we randomly varied the speed of the treadmill in the range from 1 to 5 km/h. At the beginning of the backward irregular-speed walking condition, participants could get familiar with the required unusual body movement.

While walking, participants were asked to pay attention to the passing of time and perform three different cognitive tasks of increasing difficulty. Each trial started with the instructions explaining the task to be performed from the appearance of the *Start-symbol* (2 × 2° green circle presented in the center of the screen for 800 ms) informing the participant to start estimating the passing of time, to the *End-symbol* (red circle) informing to stop temporal estimation. The least demanding task required to fixate at a small point (0.2 × 0.2°) presented on the screen center (Figure 1B). The task of medium difficulty consisted in solving easy mathematical sums of a 1-digit number plus a 2-digit number (e.g., 13+4). The result of the operations was never higher than 100. The most difficult tasks consisted in solving hard mathematical sums of a 2-digit number plus a 2-digit number with carryover (e.g., 38+49, Figure 1C). In both solve tasks, math operations (5 × 3°) were presented on a gray background and participants had 6 s to give the solutions. The experimenter pressed a key to record the response time and entered the participants answer. Then, a blank screen was presented for 1 s before the next operation (Figure 1C). At the end of each trial, after the stop symbol, a “time ruler” appeared on the screen, showing three scales of seconds from 0 to 60, one for each minute, along with the question “How much time has passed?” We use a verbal estimation paradigm, as it is the ecological way humans use to judge the passing of time and it does not interfere with body movements. Participants’ responses were typed by the experimenter on the keyboard. The time ruler has been used to promote a finer time response by participants (see Supplementary Figure 1).

Since human activities require more than just a few seconds and our interest was to test how humans estimate time in real-life situations, we test long temporal intervals. Specifically, we tested random durations along a continuum in the range of 15–120 s; the two extreme values were always presented at least once in each task, whereas the other durations to be tested were randomly selected (with integer values of seconds).

Participants performed a total of 135 trials (15 for each cognitive task, for each motor condition). The order of the motor conditions was randomly assigned: as a result, five participants performed the forward regular-speed walking first, six performed the forward irregular-speed walking first, four the backward irregular-speed walking condition first. Each experimental session was divided into nine blocks, interspersed with short breaks. At the beginning of each block, experimenters checked the participant’s body temperature. At the end of each block, experimenters asked participants to rate their level of physical and mental fatigue on a 7-point Likert scale, to ensure that participants were not under physical tiredness or attentional loss. Also, the room temperature was checked every 30 min, ensuring that it remained stable throughout the whole session.

### Data processing and statistical analysis

For each trial, we recorded the real elapsed time and the participant’s time estimation.

First, time estimates of each participant were fitted with a 2-parameter linear function (minimum least squares fit) to give individual average estimates as a function of elapsed time. Then, to measure the dependency of estimation uncertainty on duration, for each participant and condition, we calculated the residuals (root mean square errors, RMSE) of time estimation with respect to individual average estimates. At this point, for each condition, we pooled together residuals of all participants, and we averaged them in intervals of 21 s. Since the dispersion along the mean increases with time we calculated the error within each bin as RMSE*(1/$\sqrt{2*N)}$. Finally, we fitted binned data with a linear function with 2 parameters (minimum least squares fit). To assess whether RMSE increases with duration we tested whether the slopes of the fitting curves were compatible with zero (*z*-tests). To investigate whether the trends of RMSE in each task and walking condition were the same, the slopes of the fits have been statistically compared with *z*-tests.

We then calculated the time difference in seconds between estimated and effective durations (estimation bias). For each cognitive task in the three motor conditions, the estimation biases of all participants were averaged in intervals of 21 s and fitted with a 2-parameter linear function (minimum least squares fit). To assess the role of our variables in time estimation, we performed a general linear mixed-effects model (GLMM) pooling estimation biases of all participants together, with bias as the outcome variable, and cognitive task (three levels: easy, medium, hard), motor condition (three levels: forward regular-speed walking, forward irregular-speed walking, and backward irregular-speed walking) and durations (continuous) as fixed effects. We also included the variable *participants* as the random effect. Pairwise *post-hoc* comparisons between categorical factors were assessed by using *t*-tests with Bonferroni’s correction. *Post-hoc* comparisons between continuous and categorical variables were assessed with *z*-tests.

For the medium and hard tasks, we also measured correct responses and the response times for math operations, to assess possible influences of motor tasks on cognitive performance. The averaged percentages of correct responses and response times were compared with two-way ANOVA analyses, with factors; motor condition (three levels: forward regular-speed walking, forward irregular-speed walking and backward irregular-speed walking) and task difficulty (two levels: medium and hard). The *p*-values obtained from *post-hoc* analyses were adjusted using the Bonferroni correction.

To test the retrospective power of our observed effect based on the sample size and parameter estimates derived from the given data set, we performed a *post-hoc* power analysis for a general linear model (with 3 regressors) using G* Power3 (Erdfelder et al., 2009), by deriving the effect size from R-squared R^2^. Based on the consideration that simpler models are less sensitive than mixed models, we can reasonably infer that the result found with general linear model is valid also for our mixed model.

Matlab (R2018b version) and Excel (16 version) software were used for data processing, data fitting, and graphs’ creation. JASP (Version 0.8.6), G*Power (3.1.9.4), and R (4.0.3) software were used for statistical analyses.

## Results

**FIGURE 2:**
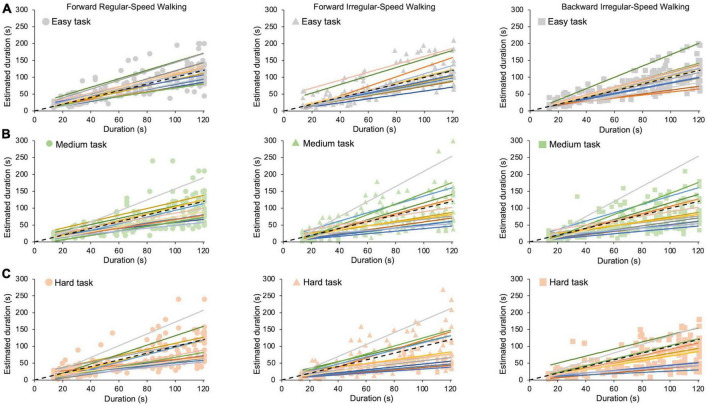
Time estimation in different motor and cognitive tasks. Each colored fit line is the average time estimate of the same participant. Dots indicate the forward regular-speed walking condition (left panels); triangles indicate the forward irregular-speed walking condition (middle panels); squares indicate the backward irregular-speed walking condition (right panels). Dashed black lines represent exact estimations. **(A)** Easy task. **(B)** Medium task. **(C)** Hard task.

In this study, participants are asked to perform cognitive tasks of different difficulties and estimate durations of up to 2 min, while being concurrently subjected to motor tasks of different difficulties. Raw data from all participants for each cognitive task (easy: looking at the screen; medium and hard: solving simple or hard sum operations) in all motor conditions (forward regular-speed walking, forward irregular-speed walking, and backward irregular-speed walking) are reported in Figure 2.

A close inspection of Figure 2 shows that participants behave very differently from each other: individual participants with high (or low) time estimates in one condition tend to maintain high (or low) estimates in all the other conditions (see different color lines in all panels). Therefore, to measure accuracy and precision, we need to take into account individual variability before averaging the data.

**FIGURE 3:**
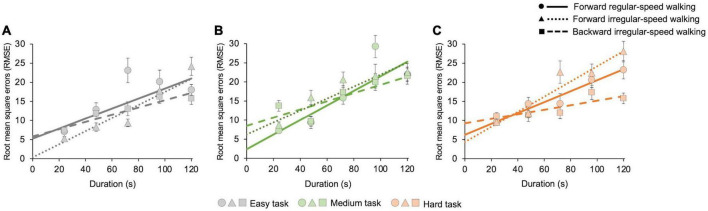
Estimation uncertainty as a function of duration. Estimation uncertainty increases with duration, similarly for each cognitive task and motor condition. The graphs show the root mean square errors (RMSE), computed on 21 s intervals, for all cognitive and motor tasks with their best-fit curves. Solid lines and dots represent the forward regular-speed walking condition; dotted lines and triangles represent the forward irregular-speed walking condition; dashed lines and squares represent the backward irregular-speed walking condition. **(A)** Easy task (number of occurrences in each bin—forward regular walking: 67, 27, 26, 23, and 82; forward irregular walking: 63, 29, 44, 37, and 52; backward walking: 69, 35, 26, 42, and 53). Goodness of fit—forward regular walking: *R*^2^ = 0.7; forward irregular walking: *R*^2^ = 0.9; backward walking: *R*^2^ = 0.9. **(B)** Medium task (number of occurrences in each bin—forward regular walking: 47, 17, 45, 49, and 66; forward irregular walking: 63, 36, 53, 24, and 49; backward walking: 48, 29, 53, 42, and 52). Goodness of fit – forward regular walking: *R*^2^ = 0.8; forward irregular walking: *R*^2^ = 0.9; backward walking: *R*^2^ = 0.7. **(C)** Hard task (number of occurrences in each bin—forward regular walking: 72, 37, 15, 53, and 48; forward irregular walking: 54, 44, 30, 46, and 51; backward walking: 64, 22, 29, 44, and 66). Goodness of fit—forward regular walking: *R*^2^ = 0.9; forward irregular walking: *R*^2^ = 0.9; backward walking: *R*^2^ = 0.7.

In Figure 3 we then reported root mean square errors (RMSE) with respect to individual average estimates. Statistics show that, for each cognitive task and motor condition, RMSE increases with duration (easy task: forward regular *z* = 2.6, *p* = 0.004; forward irregular *z* = 5.6, *p* < 0.001; backward irregular *z* = 5, *p* < 0.001; medium task: forward regular *z* = 3.8, *p* < 0.001; forward irregular *z* = 5, *p* < 0.001; backward irregular *z* = 2.5, *p* = 0.005; hard task: forward regular *z* = 14, *p* < 0.001; forward irregular *z* = 6.6, *p* < 0.001; backward irregular *z* = 3, *p* = 0.001). For each walking condition, there are no significant differences between tasks (all *p* > 0.05). We found differences between walking conditions only in the hard cognitive task (forward regular vs. forward irregular: *z* = −4.9, *p* < 0.001; forward regular vs. backward irregular: *z* = 2.6, *p* = 0.004; forward irregular vs. backward irregular: *z* = −3.9, *p* < 0.001).

**FIGURE 4:**
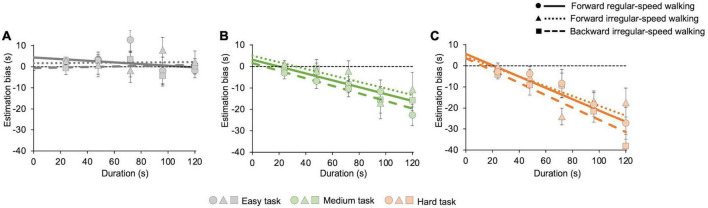
Averaged estimation bias in different motor and cognitive tasks. Underestimation increases with cognitive load but does not change with the difficulty of the motor task. The graphs show the estimation bias, computed on 21 s intervals, for all cognitive and motor tasks with their best-fit curves. Error bars are SE across participants. Solid lines and dots represent the forward regular-speed walking condition; dotted lines and triangles represent the forward irregular-speed walking condition; dashed lines and squares represent the backward irregular-speed walking condition. Dashed black lines represent exact estimations. **(A)** Easy task (forward regular-speed walking: slope = –0.04 ± 0.04, intercept = 4.4 ± 2.2; forward irregular-speed walking: slope = 0.004 ± 0.05, intercept = 1.7 ± 3.1; backward irregular-speed walking: slope = 0.01 ± 0.03, intercept = –0.6 ± 1.6). **(B)** Medium task (forward regular-speed walking: slope = –0.2 ± 0.03, intercept = 3.1 ± 1.3; forward irregular-speed walking: slope = –0.15 ± 0.05, intercept = 4.9 ± 3.2; backward irregular-speed walking: slope = –0.2 ± 0.03, intercept = 1.6 ± 1.8). **(C)** Hard task (forward regular-speed walking: slope = –0.3 ± 0.08, intercept = 5.6 ± 4.2; forward irregular-speed walking: slope = –0.2 ± 0.04, intercept = 4.2 ± 2.8; backward irregular-speed walking: slope = –0.3 ± 0.06, intercept = 3.6 ± 3.1).

Regardless of the motor condition, Figure 2 also shows that temporal estimations tend to be accurate while participants pay attention only to time (easy task, Figure 2A), whereas they perceive shorter duration while performing demanding cognitive tasks (medium and hard tasks, Figures 2B, C). The estimation biases for each task, averaged across participants, are then reported in Figure 4. We conducted a GLMM analysis after pooling estimation biases of all participants together (see Data processing below for details). First, the model reveals a main effect of the cognitive task (*χ^2^*(2) = 122.2, *p* < 0.001), confirming that the bias depends on its level of difficulty. Pairwise *post-hoc* comparisons show significant differences between all cognitive tasks (easy vs. medium: *t* = 7.7, *p* < 0.001; easy vs. hard: *z* = 11.1, *p* < 0.001; medium vs. hard: *t* = 3.2, *p* = 0.004). GLMM also reveals a significant effect of duration (*χ^2^*(1) = 104.1, *p* < 0.001), and a significant interaction between task and duration (*χ^2^*(2) = 42.9, *p* < 0.001). Underestimation increases with durations only in the medium (*z* = −7.2, *p* < 0.001, see Figure 4B) and in the hard tasks (*z* = −9.7, *p* < 0.001, see Figure 4C). No significant effect of walking emerged (*χ^2^*(2) = 2.7, *p* = 0.2), indicating that biases do not depend on the difficulty of the walking task. Marginal means and contrasts are reported in Supplementary Table 1. A *post-hoc* power analysis with effect size *f*^2^ = 0.49, alpha = 0.05, and sample size = 15 returns a power of 0.81, confirming the appropriate statistical power of the above results.

**FIGURE 5:**
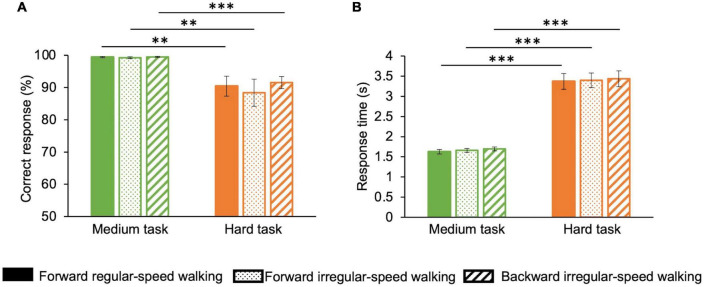
Performance for math operations in the three motor conditions. Correct responses and reaction times change with cognitive load but do not change with the difficulty of the motor task. Green: medium task; orange: hard task. Solid bars correspond to the forward regular-speed walking; dotted bars correspond to the forward irregular-speed walking; striped bars correspond to the backward irregular-speed walking. **(A)** Percentage of correct responses. **(B)** Response times. Asterisks mark statistically significant differences with ANOVAs: ^**^p < 0.01, ^***^p < 0.001. Error bars are SE across participants.

Finally, we analyzed correct responses (Figure 5A) and response times (Figure 5B) to math sums in the medium and hard tasks. In the medium task, the percentage of correct responses averaged over participants and durations is almost 100% for all walking conditions, while in the hard task, the percentage of correct responses drops to about 90% (Figure 5A). ANOVA (two factors: motor condition—three levels: forward regular-speed walking, forward irregular-speed walking, and backward irregular-speed walking—and task difficulty—two levels: medium and hard) confirms that correct responses differ across the two cognitive tasks (*F*(1,14) = 12.3, *p* = 0.003, η^2^ = 0.3) but they do not depend on the motor condition [*F*(2,28) = 0.7, *p* = 0.4, η^2^ = 0.008]. In the hard task, response times averaged over participants and durations are almost double than in the medium task for all walking conditions (Figure 5B). ANOVA confirms that response times depend on the cognitive tasks [*F*(1,14) = 122.1, *p* < 0.001, η^2^ = 0.9] but not on the motor condition [*F*(2,28) = 0.5, *p* = 0.6, η^2^ = 0].

## Discussion

Time estimation has been extensively studied for well over a century but the way in which we code temporal information is still little understood. Several pieces of evidence show that time estimation depends on many contextual variables and is distorted by concurrent cognitive and motor tasks (Block, 2003; Buhusi and Meck, 2005; Block and Zakay, 2008; Ivry and Schlerf, 2008; Baldauf et al., 2009; Wittmann, 2009; Block et al., 2010; Grondin, 2010; Sucala et al., 2010; Merchant et al., 2013; Matthews and Meck, 2014, 2016).

In our previous study (Castellotti et al., 2022), walking induced a higher estimation uncertainty and a larger underestimation of long durations only during the execution of demanding cognitive tasks, with respect to the sitting condition. We speculated that the motor task increases somehow the weight of the concurrent cognitive effort, leading to additional openings of the switch and, consequently, to a lower number of accumulated pulses than in sitting conditions (Castellotti et al., 2022). In this previous work, however, we compared a relatively automatic motor condition with a condition where motion was absent, therefore the cognitive loads due to the motor tasks were relatively lower than those due to the mental tasks and therefore not quite comparable.

For this reason, in the present study, we manipulated the difficulty of the motor task, setting an automated body movement that requires no motor or mental effort (Paul et al., 2005; Bridenbaugh and Kressig, 2011; Clark et al., 2015) (forward regular-speed walking), a more difficult movement requiring some motor control and body balance due to unpredictable speed changes (forward irregular-speed walking), and a highly demanding movement requiring continuous motor control to adapt to random speed changes (backward irregular-speed walking). We can reasonably assume that these different types of walking require a progressive increase of attentional load. We also combined these motor tasks with three cognitive tasks of increasing difficulty.

Our purpose was to investigate the influence of motor and cognitive task difficulty on time estimation. We assume that walking activity, as well as other mental tasks, need allocation of attention (Malouin et al., 2003) and therefore might affect temporal judgments with increasing distortions as a function of its difficulty, as predicted by the *attentional allocation model* (Grondin, 2010; Allman et al., 2014; Matthews and Meck, 2016). That is, walking activity could independently act on the switch to alter the number of pulses transferred to the accumulator (Gibbon, 1977; Meck, 2003; Wearden, 2003).

We first measured estimation uncertainty, finding that, according to the *scalar timing theory* (Gibbon, 1977; Meck, 2003; Wearden, 2003), it increases linearly with time. The increase of variability with duration is the same for all cognitive tasks. In the easy and in the medium cognitive tasks, uncertainties do not vary with motor conditions. In the hard cognitive task, uncertainties differ between motor conditions, being highest for forward irregular walking. This effect could be due to the higher speeds of walking involved in this condition (Karşilar et al., 2018).

We then analyzed estimation accuracy by measuring the estimation bias. The results confirm that the difficulty of the cognitive task affects estimation, with increasing underestimation with task difficulty (Polti et al., 2018; Castellotti et al., 2022). The trend of the estimation bias is also in line with previous findings: in the easy task, estimation bias remains constant across durations (Polti et al., 2018; Castellotti et al., 2022), while in harder tasks underestimation scales with durations, so that the longer durations were more underestimated than the shorter ones (Matthews and Meck, 2016; Polti et al., 2018; Castellotti et al., 2022). Time distortions induced by mental calculation might depend on the role of the right parietal cortex, which is consistently implicated in mathematical cognition (Wu et al., 2009), and time estimation (Hayashi et al., 2018). We did not find evidence of any effect of motor tasks on temporal judgments, even in the most demanding one, independently of the presence or absence of a concurrent cognitive task.

Particularly, even the continuous attention needed to perform a demanding non-automatic walking task alone does not seem to be able to alter the counting of time, as other cognitive tasks do (Brown, 1997; Polti et al., 2018). A possible explanation could be that the neural mechanisms involved in walking are not able to directly open the switch, that would produce a loss of pulses and thus a decrease in the perceived duration, as predicted by the *scalar timing theory* (Gibbon, 1977; Meck, 2003; Wearden, 2003). Alternatively, our non-automatic walking, although irregular and backward, might require a much smaller amount of attention than that needed by other mental tasks, as those involving working memory or arithmetic operations.

Since we did not find evidence of differential effects induced by diverse types of walking on temporal judgments, it has not been possible to study the nature of the interactions between motor and cognitive processes on time estimation.

The results of the current study are hard to frame in the existing literature on time estimation during motor activity. Few studies are not in line with the *attentional allocation model*, finding an overestimation of temporal durations during walking, and suggest that walking speeds up the internal clock, probably due to its physiological effects (Kroger-Costa et al., 2013; Sayali et al., 2018). However, they used non-ecological paradigms for the study of time estimation, either using very short durations (much less than 1 s; (Kroger-Costa et al., 2013)) or reproduction methods (Sayali et al., 2018). Therefore, their results are hardly comparable to ours. Other studies, instead, have found duration underestimation of short intervals (in the order of *ms*) induced by hand movements (Yokosaka et al., 2015; Tomassini and Morrone, 2016; Yon et al., 2017), suggesting that this particular motor activity is able to decrease attention to time, serving as secondary task. The difference with our results could be then explained by the fact that fine movements involve different motion circuits (Tomassini and Morrone, 2016) and probably require a larger attentional load than gross body movements like walking. In future studies then, one should use more complex motor actions (e.g., reproducing hand movements sequences), in the absence of other cognitive tasks to verify that time underestimation is replicated also at longer time intervals (in the order of *secs*), and with a concurrent cognitive task to uncover possible interaction between them.

Although cognitive performance is deteriorated by demanding postural or walking tasks (Shumway-Cook and Woollacott, 2000; Castellotti et al., 2022), here we did not find any effect of different walking types either on the number of correct responses or time responses. This result might further suggest that the manipulation introduced here, however, effective it may seem in making the motor task difficult and non-automatic, is mostly ineffective at influencing the subject’s responses.

To conclude, from our results we can infer that executing walking tasks, even demanding as walking backward at an irregular randomly-changed speed, cannot be considered in the same way as other demanding cognitive tasks, like solving hard operations (Castellotti et al., 2022) or memorizing past items (Polti et al., 2018), in distorting time estimation.

## Data availability statement

The datasets presented in this study can be found in online repositories. The names of the repository/repositories and accession number(s) can be found below: All data are available from the Zenodo database (https://doi.org/10.5281/zenodo.7492703).

## Ethics statement

The studies involving human participants were reviewed and approved by the Local Ethics Committee (“Commissione per l’Etica della Ricerca,” University of Florence, 7 July 2020, n. 111”). The patients/participants provided their written informed consent to participate in this study.

## Author contributions

SC and OD’A participated in the experiment programming, data collection, statistical analyses, and manuscript writing and review. MMDV participated in the project ideation, statistical analyses, and manuscript writing and review. All authors contributed to the article and approved the submitted version.
