# Supplementary material for: Time estimation during motor activity
Source: Front Hum Neurosci. 2023 Apr 21;17:1134027. doi: 10.3389/fnhum.2023.1134027 (PMC10160443; doi:10.3389/fnhum.2023.1134027)
Supplement: Supplementary file 1 [file Data_Sheet_1.docx]

**Supplementary Table 1.** **Marginal means and contrasts for each factor**. In the middle column, 1 indicates the forward regular-speed walking condition; 2 indicates the forward irregular-speed walking condition; 3 indicates the backward irregular-speed walking condition.

|  | Walking | Tasks | Duration |
| --- | --- | --- | --- |
| Estimated marginal means | 1 = -5.9  2 = -5.3  3 = -9.4 | Easy = 1.5  Medium = -8.9  Hard = -13.2 | = - 6.8 |
| Contrasts | 1 *vs* 2 = -11.9  2 *vs* 3 = -15.6  3 *vs* 1 = -9.4 | Easy *vs* Medium = 3.1  Medium *vs* Hard = -26.6  Hard *vs* Easy = -13.2 | - |


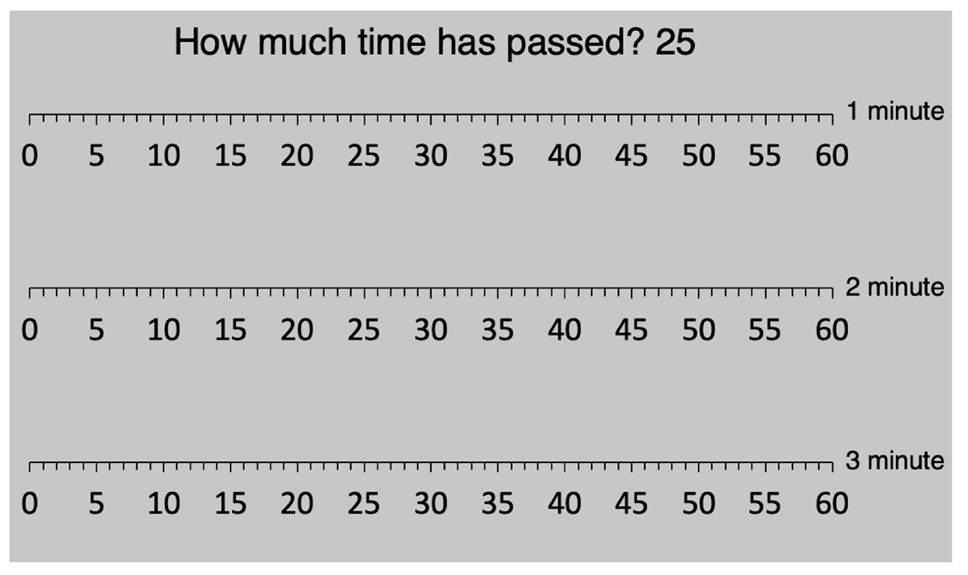


**Supplementary Figure 1. Time ruler.** Ruler shown at the end of each trial to allow participants to express how much time had passed while they were performing the task. In the example shown, the participant estimated 25 s.
